# Identification of a novel *FRMD7* splice variant and functional analysis of two *FRMD7* transcripts during human NT2 cell differentiation

**Published:** 2011-11-17

**Authors:** Yingzhi Li, Jiali Pu, Zhirong Liu, Shanhu Xu, FanYing Jin, Lijun Zhu, Jun Tian, Jianhong Luo, Baorong Zhang

**Affiliations:** 1 Department of Neurology, Second Affiliated Hospital, Zhejiang University School of Medicine, Hangzhou, China; 2 Department of Neurology, Zhejiang Hospital, Hangzhou, China; 3 Department of Neurobiology, Institute of Neuroscience, Zhejiang University School of Medicine, Hangzhou, China

## Abstract

**Purpose:**

FERM domain containing protein 7 (*FRMD7*) mutations are associated with X-linked idiopathic congenital nystagmus (ICN). The purpose of this study is to identify a novel splice variant of *FRMD7* (*FRMD7-S*) in both humans and mice with a shortened exon 4 relative to the original form of *FRMD7* (*FRMD7-FL*),and to detect the role of *FRMD7-FL* and *FRMD7-S* in the process of neuronal development.

**Methods:**

The splice variant of *FRMD7* was identified by PCR. Expression levels of h*FRMD7-FL* and h*FRMD7-S* transcripts in developing human fetal brain were tested by RT–PCR, and expression levels in the human pluripotent embryonic carcinoma NTera 2/cl.D1 (NTERA-2; NT2) cell line with all-trans retinoic acid (ATRA) or bone morphogenetic protein-2 (BMP-2) treatment were tested by real-time qPCR. hemaglutinin (HA)-tagged recombinant plasmids DNA encoding h*FRMD7-FL* and Myc-tagged recombinant plasmids DNA encoding h*FRMD7-S* were used to transiently transfect the human NT2 cells. Further, immunofluorescence experiments were performed to determine the co-localization of the two fusion proteins. Finally, using co-immunoprecipitation analyses, we demonstrated that FRMD7-FL and FRMD7-S interacted with each other.

**Results:**

A novel splice variant of *FRMD7* (*FRMD7-S*) with a shortened exon 4 relative to the original form of *FRMD7* (*FRMD7-FL*) was identified from the cDNA of the human NT2 cell line and mouse fetal brain. The *FRMD7* transcripts showed similar tissue distributions and were upregulated following all trans retinoic acid (ATRA)-induced differentiation of NT2 cells. FRMD7-FL and FRMD7-S co-localized and co-immunoprecipitated with each other. Further, overexpression of *FRMD7-FL* in NT2 cells resulted in altered neurite development and upregulation of *FRMD7-S*.

**Conclusions:**

Although the significance of the 45 bp deletion remains unknown, our observations suggest that the *FRMD7* isoforms may play a significant role during neuronal differentiation and development.

## Introduction

Idiopathic congenital nystagmus (ICN) is an oculomotor disorder characterized by involuntary horizontal oscillations of the eyes that presents at birth or appears in the first months of life, but does not usually worsen over time. ICN is distinct from other ocular disorders in which nystagmus is acquired later in life (e.g., cataracts, glaucoma, albinism) or is accompanied by eye, brain, or other health abnormalities [1]. The prevalence of ICN is estimated to be 24 per 10,000 [2], and although some techniques can improve vision (e.g., glasses, contact lenses, eye muscle surgery), nystagmus is usually permanent and cannot be corrected or cured [3].

Previous studies have speculated that ICN represents a primary defect in the brain regions involved in ocular motor control [4], although the precise pathogenic mechanisms underlying ICN are currently unknown. Mutations in the human FERM domain containing protein 7 (*FRMD7*) gene (NM_194277), which encodes the FERM domain containing protein 7 that is a member of the FERM family, are associated with X-linked ICN [5]. Approximately 50% of X-linked pedigrees and 5% of sporadic ICN cases have been linked to *FRMD7* mutations, and more than 35 *FRMD7* mutations have been reported worldwide in families with X-linked ICN from various ethnic backgrounds [1,6,7].

The 298 amino acid FERM domain was originally identified in protein 4.1 [8], and subsequent studies reported the structural, transport, and membrane-localizing functions of this domain [9-11]. Notably, the hFRMD7-FL protein is highly homologous to FARP1 (FERM, RhoGEF, and pleckstrin domain protein 1) and FARP2 proteins, which are known to play significant roles in neuronal development. FARP1 is necessary and sufficient for promoting lateral motor column dendritic growth, and FARP2 is a key molecule involved in the response of neuronal growth cones to class-3 semaphorins [12,13]. Moreover, recent studies have demonstrated that inhibition of *FRMD7* expression in mouse neuroblastoma cell line (NEURO2A) during neuronal differentiation is associated with significant delays in neurite growth and disrupted F-actin/G-actin dynamics [14]. In a mouse model, *FRMD7* expression levels were low in all adult tissue samples, whereas *FRMD7* expression levels were higher in embryos and underwent a sharp increase at embryonic day 18 in brain tissue [15]. These findings provide evidence that *FRMD7* plays a critical role during neuronal morphogenesis, in synapse function, and in neurite growth, but further studies will be required to uncover the precise mechanisms associated with *FRMD7* function.

The NT2 cell line, which is a human embryonic carcinoma cell line, differentiates into post-mitotic neuron-like cells following treatment with all trans retinoic acid (ATRA) [16,17] and into non-neural epithelial cell lineages following exposure to bone morphogenetic protein 2 (BMP-2) [18,19]. Therefore, NT2 cells provide an ideal model system that mimics normal neuronal differentiation in the brain according to many established criteria.

Previous studies have revealed that alternative splicing occurs much more frequently in the brain relative to other tissues [20,21], and alternative splicing is increasingly recognized for its role in neurologic disease [22,23]. In the current study, we have identified and characterized a novel *FRMD7* splice variant in humans and mice, h*FRMD7-S* (GenBank FJ717411) and m*FRMD7-S*, respectively; the splice variant contains a 45 bp truncation in the fourth exon and produces an altered form of the FERM domain. Herein, we investigate the expression patterns, role in neurite development, subcellular localization, and interactions associated with the *FRMD7-S* splice variant relative to the original *FRMD7-FL* isoform in NT2 cells.

## Methods

### Cell culture and ATRA/BMP-2-induced differentiation

Human NT2 cells (Cell Culture Center of Peking Union Medical College, Peking, China) were maintained in DMEM/F12 medium supplemented with 10% fetal bovine serum, 100 U/ml penicillin, and 100 μg/ml streptomycin. Prior to the induction of differentiation, the NT2 cells were incubated in 25 cm^2^ cell culture flasks in 4 ml of medium in a 37 °C, high humidity, 5% CO_2_ incubator. The cells were trypsinized and sub-cultured at a ratio of 1:3 twice weekly. For the time course analyses, the NT2 cells were seeded on a 3.0×10^6^/10 cm^2^ plate and treated the following day with 10 μM all-trans retinoic acid (ATRA) dissolved in DMSO [16,17] or with 50 ng/ml bone morphogenetic protein-2 (BMP-2) dissolved in normal saline [18,19]. The cells were collected at 12, 24, 36, 48, and 72 h after treatment for the early time points. For the later time points (5, 8, and 12 days), the cells were reseeded every three days in the continued presence of ATRA or BMP-2 and collected on the corresponding days.

### Collection of human and mouse fetal tissue

Freshly isolated 16-week post-conception (wpc) aborted human fetal tissues were collected from the Women's Hospital School Of Medicine Zhejiang University. The experimental procedures were approved by the Human Ethical Committee of the Zhejiang University. Mouse fetal brain tissue was isolated from 18-day mouse fetuses. A scissors was used to produce 1–3 mm^3^ pieces of tissue, which were snap frozen in cryogenic vials in liquid nitrogen and stored at −80 °C. Total RNA from each sample was prepared for subsequent RT–PCR experiments.

### RNA isolation, reverse transcription PCR, and quantitative real-time PCR analysis

Total RNA was isolated from human and mouse fetal tissues and NT2 cells using TRIZOL reagent (Invitrogen, San Diego, CA), according to the manufacturer’s instructions. Real-time quantitative PCR (RT-qPCR) assays were performed using a Light Cycler^TM^ real-time PCR thermocycler (Roche, Shanghai, China). Briefly, 1.5 μg of total RNA was reverse transcribed using oligo (dT) 12–18 with M-MLV Reverse Transcriptase (Promega, Madison, WI) according to the manufacturer's instructions. Relative levels of gene expression were analyzed using the SYBR^®^ Premix Ex Taq™ (TaKaRa, Dalian, China) using the recommended PCR conditions. The sequences of the RT-qPCR primers are shown in Table 1. The relative mRNA levels of target genes were calculated relative to glyceraldehyde-3-phosphate dehydrogenase (*GAPDH*) using the (=2^-△△C^_T_) method [24].

**Table 1:** Primer sequences.

**ID**	**Name**	**Sequence**
**Primer for RT–PCR/RT-qPCR**		
p1f	*FRMD7-FL*-f	5′-CAAAGCAGGTAAAAAATCCTAAGG-3′
p1r	*FRMD7-FL*-r	5′-ATGTGAGATACCATCAACGCTGT-3′
p2f	*FRMD7-S*-f	5′-TAACAAAGCAGGTAAAAATGGAC-3′
p2r	*FRMD7-S*-r	5′-AAGATGTGAGATACCATCAACG-3′
p3f	*GAPDH*-f	5′- CCCACTCCTCCACCTTTGAC-3′
p3r	*GAPDH*-r	5′- CATACCAGGAAATGAGCTTGACAA-3′
**Primer for cloning**		
pAf	pcDNA3.1-FRMD7-FL-HA-f	5′-CGCTAGCGCCACCATGCTACATTTAAAAGTGCAGT-3′
pAr	pcDNA3.1-FRMD7-FL-HA-r	5′-GCTCGAGTTAAGCGTAATCTGGAACATCGTATGGGTAAGCTAAAAAGTAATTACATG-3′
pBf	pcDNA3.1-FRMD7-S-Myc-f	5′-CGCTAGCGCCACCATGCTACATTTAAAAGTGCAGT-3′
pBr	pcDNA3.1-FRMD7-S-Myc-r	5′-GCTCGAGTTACAGATCCTCTTCAGAGATGAGTTTCTGCTCAGCTAAAAAGTAATTACATG-3′
pCf	pEGFP-FRMD7-FL-f	5′-CGCTAGCATGATTCCCAGAAGAT-3′
pCr	pEGFP-FRMD7-FL-r	5′-GCTCGAGAGCTAAAAAGTAATTACA-3′
pDf	pDsRed-FRMD7-S-f	5′-CGCTAGCATGATTCCCAGAAGAT-3′
pDr	pDsRed-FRMD7-S-r	5′-GCTCGAGAGCTAAAAAGTAATTACA-3′

Underlined nucleotides mark the HA-tag and Myc-tag.

### cDNA clone and plasmid construction

Human *FRMD7-FL* and *FRMD7-S* genes were isolated from NT2 cell cDNA (synthesized from the total RNA of natural NT2 cells) by PCR amplification using the following primers: 5′-ATG CTA CAT TTA AAA GTG CAG TTT-3′ and 5′-TTA AGC TAA AAA GTA ATT ACA TGG T-3′. The mouse *FRMD7-FL* and *FRMD7-S* genes were cloned from 18-day fetal mouse brain cDNA using the following primers: 5′-GTA TGG ATC CAT GCT CCA TTT AAA AGT G-3′ and 5′-AGC TAA GAA ATA ATT GCA TGG CTT TAG C-3′. PCR was performed in 25 μl of a reaction mixture containing DNA polymerase from *Thermococcus kodakaraensis *(KOD) DNA polymerase buffer and 1.25 U KOD DNA polymerase (Toyobo, Osaka, Japan). Subsequent cloning of PCR products into the pEASY-Blunt vector (TransGen Biotech, Beijing, China) was performed using the pEASY-Blunt Cloning Kit for Sequencing (TransGen Biotech). Sequence analyses were performed by the Invitrogen Company (Shanghai, China). To generate eukaryotic expression vectors for h*FRMD7* splice variants, cloned RT–PCR fragments in pEASY-Blunt were used as the template. The hFRMD7-FL cDNA was sub-cloned into pcDNA3.1(+) in frame with an NH_2_-terminal HA tag (hFRMD7-FL-HA) and pEGFP-N1 (pEGFP-hFRMD7-FL) with the pAf/pAr and pCf/pCr primer sets, respectively (Table 1). The h*FRMD7-S* gene was subcloned into pcDNA3.1(+) with an NH_2_-terminal Myc tag (hFRMD7-S-Myc) and plasmid pDsRed-N1 (pDsRed-hFRMD7-S) using the pBf/pBr and pDf/pDr primer sets, respectively (Table 1). NheI and XhoI were chosen as the cloning sites for all three vectors, and the sequences of all of the expression cassettes were verified.

### Cell transfection and immunofluorescence microscopy

For transient transfection experiments, the NT2 cells were placed on glass coverslips or cell culture flasks until they reached approximately 60% confluency and were transfected with FuGENE HD transfection reagent (Roche) at a ratio of 1 μg of DNA to 3 μl of FuGENE HD per coverslip. The cells were processed for immunofluorescence at 48 h post-transfection. For stable overexpression experiments, G418 (1.2 μg/ ml medium; Invitrogen) antibiotic selection was performed for 4 weeks. For the immunofluorescence experiments, the induced or transfected NT2 cells were washed with phosphate-buffered saline (PBS) and fixed for 15 min at room temperature in 4% paraformaldehyde (PFA) in PBS. The coverslips were washed twice with ice-cold PBS, and the cells were permeabilized with PBS containing 0.2% Triton X-100 for 10 min at 4 °C. After washing the cells in PBS three times for 5 min (each wash), a blocking solution containing PBS and 10% normal goat serum was applied for 1 h at room temperature. Afterwards, primary antibodies (monoclonal anti-Myc or anti-HA or antibodies; Abmart Inc., Shanghai, China) diluted in a blocking solution were applied overnight at 4 °C or for 1 h at room temperature. In the double-labeling experiments, the cultures were incubated simultaneously with two primary antibodies. After three washes in PBS, the secondary antibodies (Dylight 488, Dylight 594; Jackson ImmunoResearch Laboratories, Inc. West Grove, PA) were diluted 1:250 in a blocking solution and added for 1 h at room temperature in the dark. The secondary antibody solution was decanted and washed three times with PBS for 5 min each in the dark. Finally, the cells were incubated for 5 min with 0.1 μg/ml DAPI as a nuclear counterstain. After three washes, the coverslips were mounted with a drop of Antifade Mounting Medium (Beyotime Institute of Biotechnology, Suzhou, China) and stored in the dark at −20 °C or 4 °C. The cells were viewed using a Nikon eclipse inverted fluorescence microscope equipped with a Nikon digital camera (Nikon Corporation, Tokyo, Japan). Image overlays and contrast enhancement were performed using Adobe Photoshop CS2 (Adobe Systems Inc. San Jose, CA) software.

### Co-immunoprecipitation and immunoblot analyses

For co-immunoprecipitation (co-IP) experiments, NT2 cells were transiently co-transfected with 2 μg of each of the hFRMD7-FL-HA and hFRMD7-S-Myc vectors. At 48 h post-transfection, the total protein been extracted from the cells by using M-PER^®^ Mammalian Protein Extraction Reagent (Thermo Fisher Scientific Inc., Rockford, IL) following the manufacturer's instructions. Then the ProFound™ Co-Immunoprecipitation Kit (Thermo Fisher Scientific Inc.) was used for co-IP assays according to the manufacturer's instructions. Control immunoprecipitations were performed in the presence of non-specific immunoglobulins.

### Statistical analyses

All experiments were done in triplicate, and the data are represented as mean±SEM. Statistical significance between groups was determined using the Kolmogorov–Smirnov test and Student’s *t*-test, as implemented by SPSS13.0 (SPSS Inc., Chicago, IL). Results were considered to be statistically significant when p<0.05.

## Results

### Identification of a novel *FRMD7* splice variant and tissue distribution of two *FRMD7* transcripts

**Figure 1:**
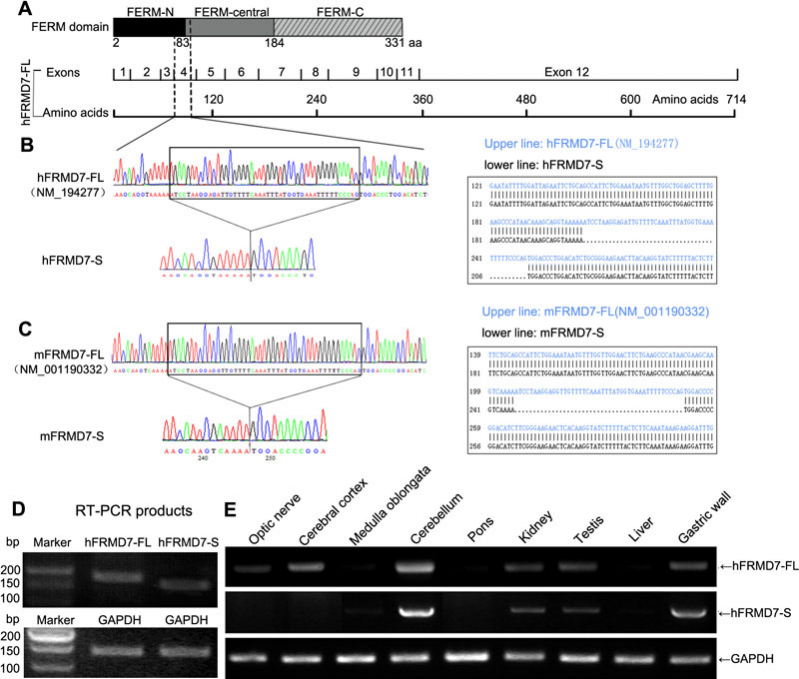
Cloning of a novel *FRMD7* isoform and tissue distribution of the two splice variants. **A**: Components of the FERM domain and gene structure of h*FRMD7-FL*. **B** and **C**: Sequence comparison between h*FRMD7-FL*/m*FRMD7-FL* (**B**) and h*FRMD7-S*/m*FRMD7-S* (**C**) showing the deletion of 45 bp in the 5′ end of exon 4 in the h*FRMD7-S*/m*FRMD7-S*. **D**: Agarose gel electrophoresis of the RT–PCR products to confirm their size and the identification of a single PCR product. E: Expression levels of h*FRMD7-FL* and h*FRMD7-S* transcripts in selected human fetal tissues. The primer sets used in **D** and **E**: p1f/p1r, p2f/p2r and p3f/p3r.

In RT–PCR experiments to clone the h*FRMD7-FL* gene from NT2 cells, two PCR products were produced. Cloning and sequence analysis of the PCR products indicated that they corresponded to two distinct *FRMD7* mRNAs: the expected original h*FRMD7-FL* mRNA and another unexpected mRNA not described previously, which is referred to herein as h*FRMD7-S*. This novel transcript, which lacks a 45 bp segment in the 5′ region of exon 4, results in the fusion of the 3′ end of exon 3 to the 5′ end of truncated exon 4; therefore, h*FRMD7-S* encodes a putative protein of 699 amino acids (Figure 1A,B). We then performed RT–PCR to confirm the presence of m*FRMD7-S* in 18-day-old fetal mice (Figure 1C). The expression of h*FRMD7-FL* and h*FRMD7-S* transcripts was examined in tissues from 16-wpc human fetal tissue using sequence-specific primers (Figure 1D). We detected high expression of h*FRMD7-FL* in the cerebellum and moderate expression in the cerebral cortex, optic nerve, kidney, testis, and gastric wall. We detected high expression of h*FRMD7-S* in the cerebellum and moderate expression in the kidney, testis, and gastric wall. However, h*FRMD7-S* was not expressed in the optic nerve or cerebral cortex. Neither h*FRMD7-FL* nor h*FRMD7-S* were expressed in the medulla oblongata, pons, or liver. Taken together, these results revealed that h*FRMD7-S* exhibited a similar but limited tissue distribution compared to h*FRMD7-FL* (Figure 1E).

### Levels of both h*FRMD7* transcripts significantly increased in ATRA-induced differentiating NT2 cells

**Figure 2:**
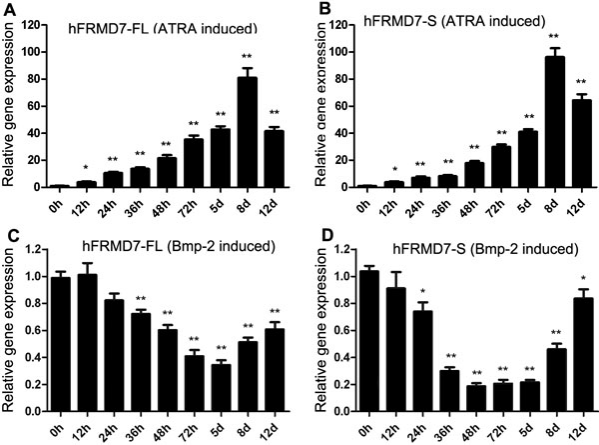
Effects of ATRA/BMP-2 induction on the expression of *FRMD7* splice variants in NT2 cells. **A** and **B**: NT2 cells were treated with ATRA (10 µM) or vehicle (0.1% DMSO) for the indicated periods. **C** and **D**: NT2 cells were treated with BMP-2 (50 ng/ml) or vehicle (normal saline) for the indicated time periods. Expression levels of h*FRMD7-FL* and h*FRMD7-S* transcripts were determined by RT-qPCR. The vehicle-treated cells did not show any significant change (data not shown). The primer sets were used for RT-qPCR: p1f/p1r, p2f/p2r and p3f/p3r. Both the transcript levels are normalized to *GAPDH* mRNA levels. The data are presented as fold changes relative to 0 h. The experiments were done in triplicate, and the graphs represent the average of three independent experiments (Columns, mean; bars, S.E.M.; *p<0.05, **p<0.01 versus 0 h).

As shown in Figure 2A,B, the levels of h*FRMD7-FL* and h*FRMD7-S* mRNA expression in NT2 cells started to increase at 12 h after ATRA treatment and reached a maximal increase at 8 days (81 fold for h*FRMD7-FL* and 96 fold for h*FRMD7-S* relative to the 0 h time point; Figure 2A,B). The control group did not exhibit any change relative to the 0 h time point. Compared to ATRA, BMP-2 had a repressive effect on h*FRMD7-FL* and h*FRMD7-S* expression. After 24 h of BMP-2 treatment, the levels of h*FRMD7-FL* and h*FRMD7-S* transcripts started to decline relative to the 0 h time point, and continued to decrease after 36 h; after 8 days, the expression levels of h*FRMD7-FL* and h*FRMD7-S* transcripts began to increase (Figure 2C,D).

### hFRMD7-FL and hFRMD7-S co-localized with class III β-tubulin and exhibited differential effects on neurite development

**Figure 3:**
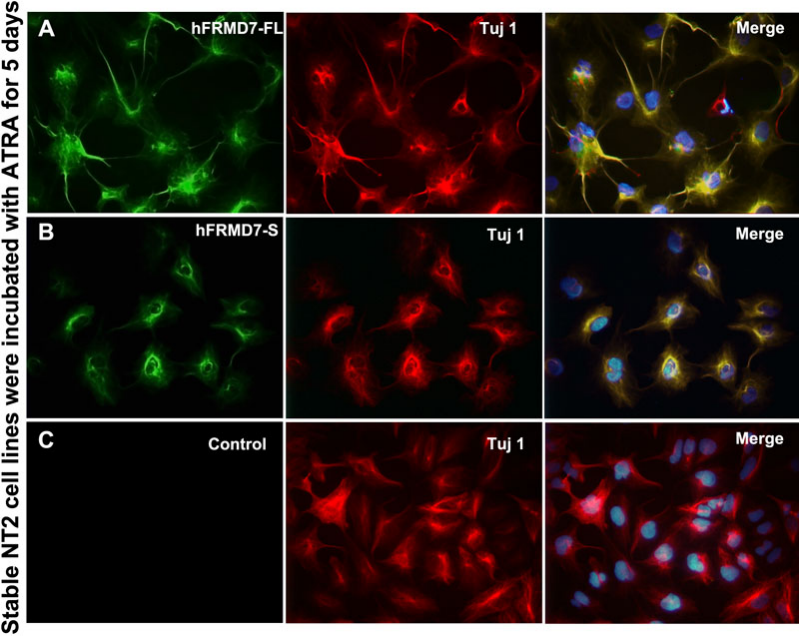
Effects of h*FRMD7-FL* or h*FRMD7-S* overexpression on neurite outgrowth. NT2 cells stably transfected with hFRMD7-FL-HA (**A**), hFRMD7-S-Myc (**B**), or the empty pcDNA3.1(+) vector (**C**) were treated with ATRA for 5 days. The cells were fixed and immunostained with monoclonal anti-Myc or anti-HA antibodies and a DyLight-488 conjugated secondary antibody. Class III β-tubulin was immunostained using a monoclonal TuJ1 antibody and DyLight-594 secondary antibody. Magnification: 200×.

Stable NT2 cell lines overexpressing hFRMD7-FL-HA or hFRMD7-S-Myc were established for use in experiments to study their role during differentiation. We examined the co-localization of class III β-tubulin, which is not expressed at detectable levels in neural progenitor cells, with the two FRMD7 isoforms by indirect immunofluorescence. Both isoforms exhibited strong co-localization with neuronal class III β-tubulin in differentiating NT2 cells induced with ATRA for 5 days (Figure 3A,B). Having established that the hFRMD7-FL or hFRMD7-S overexpressed cell lines, we next sought to find how this change would affect neurite development in differentiated NT2 cells. Notably, following ATRA treatment for 5 days, a significant increase in the length and number of neurites was observed in NT2 cells stably overexpressing hFRMD7-FL-HA NT2 (Figure 3A,C and Table 2; p<0.05). However, the degree of neurite branching did not differ between the control and hFRMD7-S overexpressing NT2 cells (Figure 3B,C and Table 2; p>0.05).

**Table 2:** Neurite outgrowth of NT2 cells treated with 10 μM retinoic acid.

**Norm**	**Control**	**hFRMD7-S**	**hFRMD7-FL**
Percentage of cells with neurites*	46.2±2.1	47.5±1.3 (ns)	77.8±1.6
Average neurite length** (μm)	42.7±2.6	43.6±2.1 (ns)	72.3±1.1

Cells were treated with 10 μm retinoic acid, 10% FCS/DMEM-F12 for 5 days, and neurite parameters were quantified. Data are mean±SEM of three independent experiments. At least 250 cells per transfection were counted. *Cells that possess at least one outgrowth of more than one-half the cell body diameter in length. **Neurite length from cell body to distal tip. (ns) stands for Not Significant.

### hFRMD7-FL co-localizes and interacts with hFRMD7-S in NT2 cells

**Figure 4:**
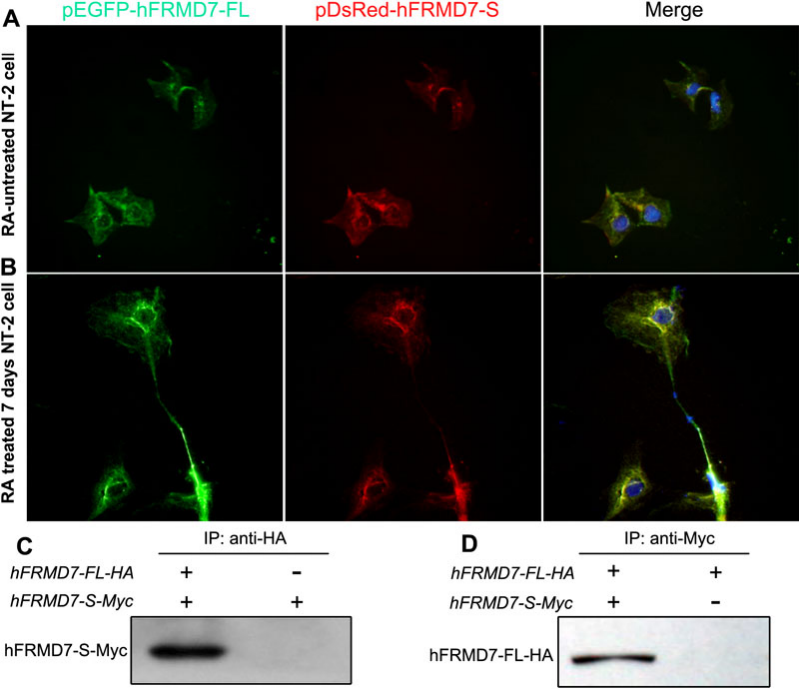
Sub-cellular co-localization and co-immunoprecipitation of hFRMD7-FL with hFRMD7-S. Co-transfection of pEGFP-hFRMD7-FL (green) and pDsRed-hFRMD7-S (red) into RA-untreated (**A**) or differentiating NT2 cells (**B**). After 48 h, the cells were fixed and DAPI was used as a nuclear counterstain (blue). Magnification 400×. **C** and **D**: Reciprocal co-immunoprecipitation experiments show that hFRMD7-FL-HA co-immunoprecipitates with hFRMD7-S-Myc, and vice versa, in NT2 cells. Cells transfected with only hFRMD7-S-Myc or hFRMD7-FL-HA served as the negative control.

To investigate the subcellular co-localization of FRMD7 isoforms and to differentiate between the localization of endogenous FRMD7 and exogenously expressed FRMD7 proteins, we used expression vectors to express hFRMD7-FL or hFRMD7-S differentially tagged with green (EGFP) or red (DsRed) fluorescent protein, respectively. Following transient co-transfection with the fusion constructs encoding pEGFP-hFRMD7-FL and pDsRed-hFRMD7-S, untreated NT2 cells (without ATRA) and NT2 cells treated with ATRA for 7 days were subjected to fluorescence microscopy. In these experiments, EGFP-hFRMD7-FL and DsRed-hFRMD7-S exhibited a high degree of co-localization (Figure 4); both isoforms were primarily localized in cytoplasm and perinuclear regions in undifferentiated NT2 cells (Figure 4A). Similarly, in NT2 cells induced with ATRA for 7 days, EGFP-hFRMD7-FL and DsRed-hFRMD7-S co-localized extensively in the cytoplasm, perinuclear regions, and neurite processes. Notably, the distribution of EGFP-hFRMD7-FL was more abundant in the distal terminals of the neurites relative to that of DsRed-hFRMD7-S (Figure 4B).

To test for protein–protein interactions, we performed co-IP experiments. We co-expressed HA and Myc NH_2_-terminally tagged versions of hFRMD7-FL (hFRMD7-FL-HA) and hFRMD7-S (hFRMD7-S-Myc), respectively, in NT2 cells. At 48 h post-transfection, total cell lysates were extracted, and hFRMD7-FL-HA was immunoprecipitated using anti-HA antibodies, followed by immunoblot analysis with anti-Myc antibodies. The results showed that hFRMD7-FL-HA co-immunoprecipitated with hFRMD7-S-Myc (Figure 4C). This finding was confirmed by reciprocal immunoprecipitation experiments, which showed that hFRMD7-S-Myc co-immunoprecipitated with hFRMD7-FL-HA (Figure 4D). The results of these experiments may suggest that hFRMD7-FL and hFRMD7-S either interact directly or are associated with the same protein complex in NT2 cells.

### hFRMD7-FL protein stimulates transcription of h*FRMD7-S*

**Figure 5:**
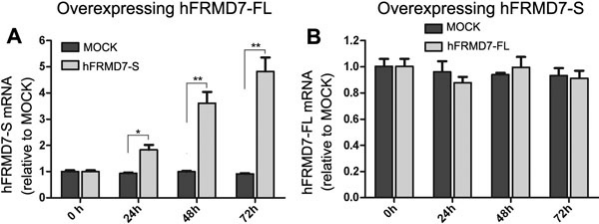
Overexpression of h*FRMD7-FL* stimulates h*FRMD7-S* transcription. Expression levels of h*FRMD7-FL* or h*FRMD7-S* transcripts were monitored by RT-qPCR in transfected NT2 cells at the indicated time points. Cells transfected with the empty vector served as the negative control (MOCK group). **A**: Expression levels of h*FRMD7-S* in NT2 cells overexpressing h*FRMD7-FL*. **B**: Expression levels of h*FRMD7-FL* in NT2 cells overexpressing h*FRMD7-S*. The primer sets were used for RT-qPCR: p1f/p1r, p2f/p2r and p3f/p3r. Both the transcript levels are normalized to *GAPDH* mRNA levels. The data are presented as the fold changes relative to MOCK group. All of the experiments were done in triplicate, and the graph represents the average (columns, mean; bars, S.E.M.; *p<0.05, **p<0.01 versus MOCK group).

Alternative splicing often impacts the transcription and subsequent translation of the major protein. Therefore, we performed experiments to determine whether overexpression of one *FRMD7* splice variant was associated with altered transcription of the other splice variant. Overexpression of h*FRMD7-FL* or h*FRMD7-S* was confirmed by RT–PCR and immunoblot analyses (data not shown). We found that the transcription levels of h*FRMD7-S* exhibited a time-dependent increase in cells overexpressing h*FRMD7-FL* relative to control cells, with an average of 4.8 fold higher h*FRMD7-S* expression levels at 72 h (Figure 5A); in contrast, h*FRMD7-FL* transcription levels did not change significantly in NT2 cells overexpressing h*FRMD7-S* (Figure 5B).

## Discussion

The human *FRMD7* gene spans a region of 2,145 bp and comprises 12 exons. Until now, no *FRMD7* mRNA variants have been described. Herein, we report the discovery of a novel transcript, h*FRMD7-S*, from the human *FRMD7* gene generated by alternative splicing. An increased understanding of the h*FRMD7-S* variant relative to the original h*FRMD7-FL* transcript may help clarify the role of *FRMD7* splice variants in neuronal development and the pathogenesis of ICN.

We found that the h*FRMD7-FL* and h*FRMD7-S* transcripts exhibit a similar tissue distribution, with highest levels in the cerebellum; neither h*FRMD7-FL* nor h*FRMD7-S* were expressed in the medulla oblongata or pons. These results corroborate previous studies in mouse models [15]. Further, our previous studies demonstrated that h*FRMD7-FL* expression is ubiquitous during fetal brain development, but is restricted to regions of eye movement in adults [25]. We may suppose that the *FRMD7* transcripts may carry out cerebellum-associated functions (e.g., vestibulo-cerebellar system regulation of normal eye movement). Nevertheless, in the previous work, using immunohistochemistry testing, *FRMD7-FL* was observed to be highly expressed in the pons and medulla oblongata. Since the size differences in the corresponding mRNAs and protein isoforms are very small (i.e., 2,145 bp for h*FRMD7-FL* versus 2,100 bp for h*FRMD7-S*), neither northern blot nor immunoblot analyses were performed to determine endogenous hFRMD7-FL and hFRMD7-S protein expression. The reason for the differences of expression distribution between RNA level and protein level of FRMD7-FL are worth exploring in future studies.

Recent studies suggest that ATRA-induced differentiation is followed by the upregulation of h*FRMD7-FL* mRNA and protein expression levels in NEURO2A cells [14]. In the current study, we found that ATRA-induced neuronal differentiation of NT2 cells resulted in elevated expression of both h*FRMD7-FL* and h*FRMD7-S* transcripts, whereas BMP-2 treatment of NT2 cells had a repressive effect on h*FRMD7-FL* and h*FRMD7-S* expression. Further, a recent study demonstrated that downregulation of h*FRMD7-FL* in NEURO2A cells resulted in altered neurite development [14]. In the current study, we found that h*FRMD7-FL* overexpression leads to a significant increase in neurite length in differentiating NT2 cells; however, h*FRMD7-S* overexpression had no effect on this process.

NT2 cells undergo programmed terminal differentiation along a neuronal pathway, resulting in the accumulation of neuronal cells with neurite outgrowths and extended processes within a period of 8 days. Thus, we speculate that both h*FRMD7-FL* and h*FRMD7-S* are involved in the process of neurite outgrowth extension. Since h*FRMD7-FL* and h*FRMD7-S* share highly similar sequences, their different effect on ATRA-induced NT2 differentiation is of particular interest. Although it is unclear what underlies these differences, our findings suggest that the *FRMD7* isoforms are involved in the formation and growth of neurites during neuronal differentiation, and also provide evidence that the h*FRMD7-S* isoform may play a minor role during this process.

A previous study showed that *FARP1* and *FARP2*, which share homology with *FRMD7*, are involved in signal transduction between the plasma membrane and cytoskeleton, and that the hFRMD7-FL protein co-localizes with actin in NEURO2A cells [12-14,26,27]. In the current study, we found that hFRMD7-FL and hFRMD7-S splice variants also exhibit strong co-localization with class III β-tubulin, which is considered to be one of the earliest neuron-associated cytoskeletal marker proteins during mammalian development [28,29]. These results give evidence that the FRMD7 isoforms carry out functions associated with microtubules and cytoskeleton-associated proteins or at least play a role in a related molecular pathway.

We found that the hFRMD7-FL isoform co-localized to a high extent with the hFRMD7-S isoform in undifferentiated and differentiating NT2 cells, although the hFRMD7-FL isoform exhibited a more abundant distribution in the distal terminals of axon branches relative to the hFRMD7-S isoform. These differences may be due to different levels of protein translation efficiency. Further, the results of our reciprocal co-IP experiments also suggest that hFRMD7-FL and hFRMD7-S interact in NT2 cells. Taken together, these results uncovered a putative protein complex formed by the two FRMD7 isoforms; however, further studies will be required to confirm this finding.

Notably, we also found that h*FRMD7-FL* overexpression led to the stimulation of h*FRMD7-S* gene expression in NT2 cells, whereas overexpression of h*FRMD7-S* showed no significant effect on h*FRMD7-FL* expression. These results indicate that h*FRMD7-FL* might be involved in h*FRMD7-S* regulation. Considering their different effects on ATRA-induced NT2 differentiation, we speculate that h*FRMD7-S* plays a less important role of biologic functions. Although the mechanism(s) by which *FRMD7-FL* regulates h*FRMD7-S* expression remains elusive, we speculate that the altered FERM domain in hFRMD7-S may explain its inability to regulate h*FRMD7-FL* expression.

In conclusion, we identified a novel *FRMD7* variant generated by alternative mRNA splicing that results in the deletion of 45 base pairs in exon 4. This is the first splice variant of *FRMD7* to be reported. The two isoforms share a similar tissue distribution, and they co-localize and interact with each other in NT2 cells. However, the hFRMD7-S isoform showed a slightly limited tissue distribution and a limited function in altering the length and number of neurites in differentiating NT2 cells relative to the hFRMD7-FL isoform. Taken together, although the significance of the 45 bp deletion remains unknown, our observations suggest that the FRMD7 isoforms may play a significant role during neuronal differentiation and development. Therefore, although additional studies will be required to further confirm the association between the two transcripts, the current findings may enhance our understanding of their precise functional roles in the pathogenesis of ICN.
